# Genetic and environmental risk factors of asthma: a narrative review

**DOI:** 10.3389/fimmu.2026.1815798

**Published:** 2026-05-28

**Authors:** N.K. Altynova, L.P. Lebedeva, M.S. Abdullayeva, A.M. Kassymbekova, K. Yergali, N. Homaira, L.B. Djansugurova

**Affiliations:** 1 Laboratory of Population Genetics, Institute of Genetics and Physiology, Almaty, Kazakhstan; 2 Department of Genetics, Al-Farabi Kazakh National University, Almaty, Kazakhstan; 3 School of Clinical Medicine, UNSW Medicine & Health, Sydney Children’s Hospital, Sydney, NSW, Australia

**Keywords:** air pollution, asthma, external factors, gene-environment interactions, genetic predisposition, GWAS, PM2.5

## Abstract

Asthma is a heterogeneous and polygenic disease, with numerous genes involved in immune regulation and respiratory epithelial function. However, currently identified genetic variants explain only part of asthma heritability. Environmental factors including smoking, stress, obesity, diet, occupational exposure, respiratory viral infections, early-life allergen exposure, low physical activity and air pollution (PM_2.5_) play an important role in disease development. The literature results also suggest that exposure to PM_2.5_ may increase the risk of asthma, particularly in genetically susceptible individuals. The aim of this narrative review was to provide an overview of environmental and genetic factors associated with asthma and to analyze the role of genetic risk variants and fine particulate matter (PM_2.5_) in the development of the disease. This review summarizes the current evidence on genetic and environmental factors involved in both the development of bronchial asthma and the determination of its severity, with an emphasis on their interaction

## 1 Introduction

Asthma has been documented in medical literature since antiquity, yet for much of its history it was studied as a functional respiratory disorder rather than as an inflammatory disease. A fundamental shift in our understanding of asthma occurred in the mid-20th century, driven by clinical, pathomorphological, and immunological studies (1–5). That reconceptualization remains the basis of current understanding: asthma is now defined by chronic airway inflammation, recurrent episodes of wheezing, coughing, shortness of breath, chest tightness and bronchial hyperresponsiveness, with symptoms that vary in severity and are typically associated with reversible airway obstruction (6–8). Common long-term adverse effects of asthma include airway remodeling, including hypertrophy and hyperplasia of smooth muscle cells, epithelial damage with thickening of the basement membrane, excessive collagen deposition in the extracellular matrix, and transformation of fibroblasts into myofibroblasts (9).

Epidemiological data indicates a high global prevalence of asthma. Although asthma mortality decreased by more than 50% between 1990 and 2019, there were approximately 262 million cases worldwide in 2019, resulting in 455, 000 deaths, most of which were preventable (10, 11). By 2021, global prevalence had reached an estimated 3, 340 cases per 100, 000 population, and age-standardized rates are projected to continue rising through 2050 (12). The prevalence of asthma varies widely (1-29%) depending on region and age, with the highest rates observed in children and adolescents. In adults, asthma is less prevalent but is characterized by a greater burden of late-onset and clinically severe forms, particularly in low and middle-income countries (13). Data from the ISAAC and Global Asthma Network (GAN) studies reveal significant heterogeneity in symptom trends: prevalence continues to rise in African and Eastern Mediterranean countries, remains stable across most of Europe and the Americas, and has declined in parts of Southeast Asia (14). The Global Initiative for Asthma (GINA) forecasts that the number of asthma patients could reach 300 million by 2025, with daily mortality reaching up to 1, 000 people (6).

**Figure 1:**
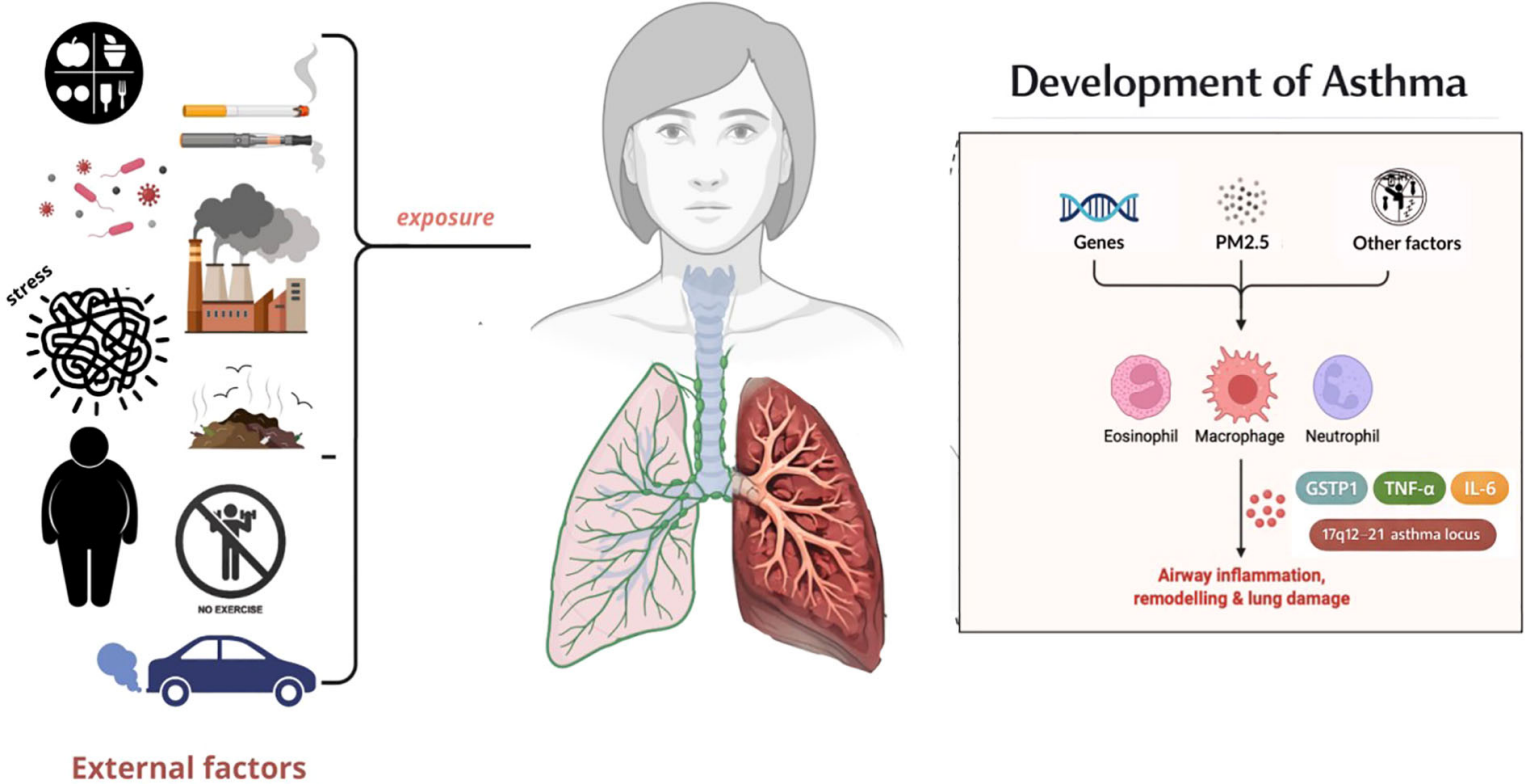
Interaction between genetic susceptibility and environmental exposures in asthma.

Asthma is a multifactorial disease that emerges from the interaction of external and internal risk factors (**Figure 1**). External factors include allergen exposure, tobacco smoke, viral respiratory infections, medications such as aspirin and non-steroidal anti-inflammatory drugs, occupational exposure to high and low-molecular-weight chemical compounds, chronic psychosocial stress, and ambient air pollution (5, 15–20). Among these, fine particulate matter (PM_2.5_) particles with a diameter of 2.5 μm or less is of particular concern given its global ubiquity and the specificity of its effects on airway epithelium. Long-term exposure to PM_2.5_ is associated with a significant higher risk of asthma and may account for approximately one-third of cases worldwide; children exposed to higher PM2.5 concentrations are more likely to develop both asthma and persistent wheezing compared to those with lower exposure (5, 21, 22). Importantly, adverse effects can occur even at relatively low pollution levels in individuals with genetic susceptibility or sensitivity.

Internal risk factors include genetic predisposition, characteristics of innate and adaptive immune responses, obesity, preterm birth, deficiencies in vitamins D, C, and E, micronutrient imbalances, particularly zinc, dysregulation of omega-3, omega-6, and omega-9 polyunsaturated fatty acids during early childhood and comorbid conditions such as rhinitis, chronic sinusitis and gastroesophageal reflux disease. Twin and family studies estimate that heredity contributes 55-74% of asthma risk in adults, with heritability varying by phenotype and age of onset (3, 23–26). Genome-wide association studies (GWAS) and large-scale meta-analyses have confirmed the pronounced polygenic architecture of asthma, with hundreds of associated loci identified (22, 27, 28). Nevertheless, identified variants account for only a fraction of asthma’s heritability and the mechanisms by which genetic background modulates environmental susceptibility remain incompletely understood.

Environmental exposures also operate through epigenetic mechanisms, including DNA methylation, histone modification and non-coding RNAs that regulate gene expression without altering the underlying DNA sequence (29). These mechanisms provide a plausible biological pathway through which exposures such as PM_2.5_, tobacco smoke, diet, the microbiome and allergens modulate immune and inflammatory pathways relevant to asthma. Epigenome-wide association studies (EWAS) have demonstrated that ambient PM_2.5_ is associated with small but consistent methylation changes in asthma-relevant tissues including blood, nasal epithelium and cord blood (30, 31). Other studies additionally show that diesel particles and allergens together upregulate TET1 and DNMT1, reshaping methylation patterns in oxidative stress and immune genes alongside coordinated histone modifications at promoters and enhancers (29). However, the observed per-exposure methylation differences are small (≤0.1-1%), and epigenetic signatures are highly tissue and cell-type specific and subject to exposure misclassification, complicating causal interpretation (31).

The extent to which genetic risk, lifestyle and gene-environment interaction jointly determine PM_2.5_-related asthma risk remains an open question. Following a historical overview of asthma from symptomatic description to its current understanding as a heterogeneous inflammatory disease, this review examines the evidence on genetic predisposition and the effects of fine particulate matter on asthma development and severity. Other environmental and lifestyle factors including smoking, diet, obesity, stress, and infections are discussed to provide biological and clinical context for gene-environment interactions. Gene interaction pathways are further examined to clarify the biological basis of asthma susceptibility.

### 1.1 Heterogeneity of asthma

Asthma is a heterogeneous respiratory disease comprising various clinical phenotypes and molecular endotypes. These subtypes differ in their inflammatory mechanisms and therapeutic responses and are broadly classified as type-2-high (T2-high) or type-2-low (T2-low). T2-high asthma is characterized by elevated levels of *IL-4, IL-5*, and *IL-13*, along with significant eosinophilic infiltration. In contrast, T2-low asthma includes neutrophilic or pauci-granulocytic inflammation involving *IL-17, IFN-γ*, and other mediators distinct from the classical Th2 response (32–35).

The introduction of allergens, respiratory viruses, and air pollutants into the respiratory tract can damage the epithelium. In response, damaged epithelial cells release cytokine alarmins, such as thymic stromal lymphopoietin (*TSLP*), interleukin-33 (*IL-33*), and interleukin-25 (*IL-25*), which function as upstream regulators of the type 2 immune response. These alarmins activate innate immune cells and promote Th2 polarization, ultimately leading to IgE production and the activation of mast cells and basophils (36). Activated mast cells then release proinflammatory mediators, including histamine, mast cell mediators, prostaglandins, and leukotrienes, which trigger the early phase of bronchospasm by inducing smooth muscle contraction, increasing vascular permeability, and stimulating mucus secretion (37, 38).

Epithelial mediators activate dendritic cells and ILC2s, which initiates a Th2-mediated immune response. This response involves the production of *IL-4, IL-5*, and *IL-13*, ultimately leading to IgE-mediated allergic sensitization, eosinophilic inflammation, epithelial damage, and persistent bronchial hyperresponsiveness (39).

Classic allergic asthma typically presents with early-onset symptoms, high IgE levels, and atopy. In contrast, late-onset asthma often exhibits more complex inflammatory profiles and a weaker association with classic allergic mechanisms, highlighting the importance of considering the age of onset in asthma phenotyping. Asthma phenotypes are further classified using various clinical parameters, including age of onset, severity and duration of the condition, frequency of acute exacerbations, respiratory dysfunction, level of symptom control, biomarkers, and treatment response, including potential hormonal resistance (40). Beyond the classic Th2-mediated pathway, other asthma phenotypes exist, such as non-allergic, neutrophilic, and Th17-associated variants, in which innate immune mechanisms and responses to air pollutants or infections are significant (41). In children, viral infections, particularly those caused by rhinoviruses and respiratory syncytial virus (RSV), can damage the epithelium and disrupt its repair processes, contributing to persistent airway hyperresponsiveness (42). According to the latest GINA 2025 data, there are seven recognized asthma phenotypes, including exercise-induced asthma, cough-variant asthma, and asthma with obesity, in addition to those previously mentioned (43, 44).

The development of bronchial asthma is a multifaceted process involving epithelial damage, immunological sensitization, Th2-mediated inflammation, and airway remodeling. The combined action of these mechanisms results in the clinical heterogeneity of the disease, variable therapeutic responses, and diverse patterns of individual disease progression.

The studies included in this review assess asthma severity in different ways: clinical classification according to GINA reports, inflammatory phenotype-based categorization, airway remodeling assessments, and cytokine-mediated mechanistic analyses. Environmental exposures, particularly air pollution and chronic psychosocial stress have been independently associated with an increased risk of severe exacerbations, revealing that disease severity is not solely a function of biological endotype but reflects a complex relationship between intrinsic and extrinsic determinants.

Several non-genetic factors have been consistently linked to asthma development and progression, including tobacco smoke, chronic stress, obesity, malnutrition, occupational exposures, infections, and allergens.

## 2 Non-genetic determinants of asthma

### 2.1 Infectious and allergen-related factors

#### 2.1.1 Early viral infections

Early viral respiratory infections occupy a central role in both the initiation and severity of asthma. Respiratory syncytial virus and rhinovirus are among the best-characterized triggers, capable of triggering asthma onset through dysregulation of antiviral immunity, particularly reduced interferon responses alongside activation of epithelial-derived cytokines and type-2 inflammatory pathways including *IL-4, IL-5*, and *IL-13* (35). Bacterial pathogens, including *Haemophilus influenzae*, *Streptococcus pneumoniae*, *Mycoplasma pneumoniae* and *Chlamydia pneumoniae*, may further amplify airway inflammation. Individuals with asthma frequently exhibit impaired innate and adaptive immune responses, including immunoglobulin deficiencies, that leave individuals more vulnerable to respiratory infections and contribute to higher exacerbation rates (35).

The clinical importance of these associations has been demonstrated across some study designs. In the Childhood Origins of Asthma (COAST) birth cohort a prospective study of 289 children at high atopic risk followed from birth to age 6 found that rhinovirus-induced wheezing illnesses in the first three years of life were the single strongest predictor of asthma at school age, regardless of aeroallergen sensitization or other risk factors; infants affected by rhinovirus-associated wheezing faced up to a 10-fold increased asthma risk, compared with only 16% of children without early wheezing episodes (45). Kusel et al. extended these findings in a community-based Western Australian cohort of 198 children at high atopic risk, followed from birth to age 5. Severe lower respiratory infections in the first year of life were most strongly linked to persistent asthma when they occurred alongside early atopic sensitization at or before age 2, an association absent in non-atopic children or those sensitized later. This points to a narrow developmental window in which viral infection and allergic sensitization together increase the asthma risk (46). Jartti and Gern reviewed this body of evidence and confirmed that severe illness caused by either RSV or rhinovirus is associated with later asthma development, with the greatest risk in young children who wheeze specifically with rhinovirus, while also noting that whether viral illnesses directly cause asthma or merely mark pre-existing susceptibility remains a subject of active debate (47).

#### 2.1.2 Early, intense exposure to inhaled allergens

Allergic sensitization is a major contributor to asthma risk, particularly in children. It occurs when the immune system develops IgE-mediated responses to environmental antigens that are otherwise harmless to most individuals. Common sensitizing allergens include pollen, house dust mites, animal dander, certain foods and insect venom. The relationship between allergic disease and asthma is bidirectional: allergic sensitization predisposes to asthma, and inflamed airways are in turn more vulnerable to allergen-triggered exacerbations (48).

In a cross-sectional observational study of 1, 700 Saudi schoolchildren from primary, intermediate, and secondary schools in Najran, southwestern Saudi Arabia (49) used the Arabic ISAAC questionnaire and standardized skin prick testing to assess allergic disease prevalence and risk factors. Physician-diagnosed asthma, allergic rhinitis and atopic dermatitis were found in 27.5%, 6.3%, and 12.5% of children, respectively. Multivariate analysis identified male sex (aOR 1.27), fast food consumption (aOR 1.53), proximity to heavy truck traffic (aOR 1.86), and dog or cat ownership (aOR 1.85) as significant independent risk factors. Overall, 42.5% of children (n=722) tested positive to at least one allergen on skin prick testing, with grass pollen (60%), cat hair (41.6%), and house dust mites (25%) as the leading sensitizers. Prospective studies further confirm that early, intense exposure to inhaled allergens including house dust mites, cockroaches, pollen, and animal dander combined with early allergic sensitization, significantly raises the risk of asthma development (50, 51).

### 2.2 Lifestyle and behavioral factors

#### 2.2.1 Smoking

Smoking is a well-established risk factor for asthma that contributes to both disease development and severity. Inhaled tobacco smoke contacts airway epithelium, a tissue with dual barrier and immune functions that secretes cytokines and chemokines, thus, triggering inflammatory responses, increasing membrane permeability, altering key gene expression and ultimately promoting tissue remodeling. This occurs through oxidative stress, epithelial damage, and dysregulation of both innate and adaptive immune responses, including enhanced type-2 airway inflammation (21).

Meta-analyses demonstrate that prenatal and passive tobacco smoke exposure increases asthma risk in children by 21-85%, with the strongest effects observed for prenatal exposure in children aged ≤2 years (OR = 1.85, 95% CI 1.35-2.53) (52, 53). In adults both past and current active smoking are significantly associated with asthma risk; former smokers exhibit particularly elevated risk compared to current or never-smokers (16, 54).

Miyake et al. (16) analyzed data from 75, 411 mother-child pairs in the Japanese Environmental and Children’s Study (JECS) using logistic regression and found that children whose mothers continued to smoke during pregnancy had a significantly increased risk of asthma by age 3 (OR = 1.34, 95% CI 1.15-1.56). Elevated risk was also observed among children of mothers who quit before (OR = 1.09, 95% CI 1.02-1.18) or after (OR = 1.11, 95% CI 1.01-1.23) announcing their pregnancy, suggesting lasting epigenetic or developmental consequences of even brief gestational tobacco exposure. Smoking further contributes to asthma severity by increasing exacerbation frequency and impairing lung function through sustained oxidative stress and immune dysregulation.

#### 2.2.2 Excess body weight and obesity

Excess body weight and obesity are independent, dose-dependent risk factors for asthma in both children and adults. A meta-analysis of seven prospective cohort studies (n=333, 102 participants) found that overweight and obesity (BMI ≥25) increased asthma odds compared to normal weight (OR 1.51, 95% CI 1.27-1.80), with a clear dose-response: overweight individuals had an OR of 1.38 (95% CI 1.17-1.62) and obese individuals an OR of 1.92 (95% CI 1.43-2.59; p<0.0001 for trend) (55). This association was consistent across sexes, with ORs of 1.46 (95% CI 1.05-2.02) in men and 1.68 (95% CI 1.45-1.94) in women. A prospective analysis of a Finnish cohort (n=60, 639 men and women aged 25–74 years) found that overweight was associated with ORs of 1.34 (95% CI 1.24-1.43) in women and 1.25 (95% CI 1.14-1.37) in men; for obesity, these increased to 1.57 (95% CI 1.44-1.71) and 1.63 (95% CI 1.44-1.83), respectively (56). The associations were independent of smoking, height, and physical activity, and overweight/obesity was estimated to account for 30.8% of asthma cases in women and 19.2% in men.

Obesity shifts the immune environment toward elevated Th1 and Th17 activity, whereas classic allergic asthma is driven primarily by Th2 responses. When children with asthma alone are compared to those with both obesity and asthma, the latter show higher peripheral CD4+ Th1 cell concentrations, while Th2 levels remain unchanged; their sputum also contains more neutrophils and fewer eosinophils, reflecting a less typical, non-eosinophilic inflammatory profile (57). These immune cell differences carry functional consequences: Th1 cells produce IFN-γ, which drives macrophage activation and amplifies cellular immunity, while Th17 cells release pro-inflammatory *IL-17A* and *IL-21*, cytokines whose dysregulation is also implicated in autoimmune conditions such as psoriasis, multiple sclerosis, and rheumatoid arthritis (58). In the context of asthma, this obesity-driven shift toward Th1 and Th17 dominance may contribute to a more severe disease course and reduced responsiveness to standard corticosteroid treatment.

#### 2.2.3 Diet

Although none of the primary studies included in this review specifically examined dietary factors as an independent variable, diet has been consistently discussed as a relevant contributor to asthma risk across reviews and meta-analyses. An unbalanced diet typically characterized by low intake of fresh fruits and vegetables alongside excess consumption of thermally processed foods. It raises systemic oxidative stress and promotes subsequent inflammation (59). This occurs because processed food-driven oxidative stress elevates reactive oxygen species (ROS), which in turn damage DNA, proteins, lipids, and other cellular components; when sustained, this cascade creates conditions favorable to respiratory disease, including asthma (60). In other words, diet does not act on airway biology directly but through a shared oxidative and inflammatory pathway that interacts with other environmental risk factors discussed in this review.

Seyedrezazadeh et al. (61) analyzed 38 studies published between 1990 and 2013 and found that high fruit consumption (OR = 0.78, 95% CI 0.70-0.87) and high vegetable consumption (OR = 0.86, 95% CI 0.75-0.98) were each associated with reduced risk of asthma and wheezing in both adults and children, though no association was identified for dietary intake during pregnancy. Papamichael et al. (62) demonstrated that a Mediterranean diet supplemented with two weekly servings of fatty fish may serve as a practical strategy for reducing airway inflammation in childhood asthma. In a broader work, Antonogeorgos et al. (63) confirmed an inverse relationship between adherence to Mediterranean or fruit and vegetable-rich dietary patterns and childhood asthma onset. A high-fiber diet also shapes gut microbial activity, promoting the production of short-chain fatty acids (SCFAs). In animal models, maternal high-fiber or acetate intake has been shown to epigenetically program offspring lungs through histone deacetylase inhibition and altered fetal gene expression to resist allergen-induced inflammation. While human data remain limited, higher maternal fiber intake and elevated infant fecal SCFA levels have both been associated with reduced childhood asthma risk (64).

### 2.3 Low physical activity and adverse behavioral factors

Low physical activity is associated with increased asthma risk, partly mediated through obesity, chronic systemic inflammation, and impaired airway epithelial barrier function, mechanisms that amplify bronchial hyperresponsiveness (65–67). A cross-sectional analysis by Eikemans et al. (65) synthesized available data on the physical activity-asthma relationship and suggested that higher activity levels may be associated with modest protection against asthma. However, most available studies are cross-sectional, which means causality cannot be established. It remains possible that early asthma symptoms themselves lead to reduced physical activity, rather than low activity contributing to asthma. Longitudinal studies using objective activity assessment in prospective cohorts are needed before definitive conclusions can be made.

### 2.4 Chronic psychosocial stress

Chronic psychosocial stress is a risk factor for asthma development and exacerbation, operating through neuroendocrine and immune pathways that amplify airway inflammation and increase sensitization to allergens and irritants (68, 69) Prenatal stress is of particular concern: by altering the Th1/Th2 cytokine balance toward a persistent Th2 response, prenatal stress may disrupt immune, hormonal and epigenetic fetal programming as well as lung developmental processes, thereby elevating childhood asthma risk (70). Evidence from cross-national analyses spanning North America, South America, Europe and Asia further suggests that ethnicity, sex, and ambient air pollutants can modify the magnitude of this prenatal stress effect (68).

At the individual level, a Finnish adult cohort study demonstrated that exposure to multiple adverse childhood experiences significantly increased adulthood asthma risk (71). In the Tucson Children’s Respiratory Study, children without an asthma diagnosis by age 16 but with high life-event scores were four times more likely to develop asthma by age 29 (RR = 4.07, 95% CI 1.33-12.43, p=0.014) (72). However, findings are not consistent: a pooled individual-level analysis of 102, 175 working-age adults across 11 European studies (1985–2010) found that while stress can trigger asthma, work-related stress alone in the absence of broader life stressors was not associated with severe exacerbations at the population level (73) This suggests that the asthma-promoting effects of stress may require co-exposure to other stressors or operate primarily in vulnerable subgroups.

### 2.5 Environmental and occupational exposures

#### 2.5.1 Occupational exposures

Occupational exposures account for up to 20% of adult-onset asthma cases. High-molecular-weight sensitizers typically cause IgE-mediated occupational asthma, whereas low-molecular-weight compounds such as acid anhydrides can induce both immunological and non-immunological disease mechanisms (74–76).

Talini et al. (77) examined 1, 289 asthmatic individuals aged 15–46 years in Tuscany, Italy and found that 41% held occupations with recognized exposure risk, while 48.6% reported contact with gases, dust or vapors. Prevalence of both work-related and occupational asthma was higher among those with high exposure levels, and was associated with more severe disease and poorer asthma control. A review by Del Roio et al. (78) further emphasized the diagnostic importance of distinguishing occupational asthma caused by sensitizers or irritants from work-exacerbated asthma, noting that accurate differentiation is essential for timely treatment, prevention of further exposure and appropriate social and occupational counseling. Work-related asthma represents a preventable public health burden with significant long-term health and socioeconomic consequences (79).

#### 2.5.2 Air pollutants

Air pollution is an environmental risk factor for both asthma incidence and exacerbation. High outdoor concentrations of particulate matter (PM) and gaseous pollutants including nitrogen dioxide (NO_2_), ozone (O_3_), and sulfur dioxide (SO_2_) have been associated with increased asthma risk in children and adults (5, 8, 21). PM_2.5_ is considered the most biologically active component of this mixture - its small size allows it to penetrate deep into bronchioles and alveoli and has been widely linked to respiratory disease (67).

PM_2.5_ originates from both natural sources: dust, forest fire soot, volcanic ash and anthropogenic emissions including vehicle exhaust, fuel combustion and construction activity (80, 81). Its composition includes polycyclic aromatic hydrocarbons (PAHs) formed by incomplete combustion of organic materials, which are recognized carcinogens. Epidemiological surveillance data from Li et al. indicate that mean PM_2.5_ exposure across 15 studies ranged from 9.6 to 66.5 μg/m³, while NO_2_ ranged from 8.4 to 29.3 μg/m³ and O3 from 41.2 to 78.1 μg/m³ (82). Concentrations between 8 and 20+ μg/m³ have been associated with increased cardiovascular risk, including 16% increased ischemic heart disease mortality and 14% increased stroke mortality (82, 83), as well as increased risk of dementia and psychiatric disorders and contributions to multi-organ dysfunction (80–86). Globally, approximately two billion people, roughly one quarter of the world’s population live in areas with PM_2.5_ concentrations exceeding 50 μg/m³ (87, 88).

Air pollutants contribute to asthma through several biological mechanisms: oxidative stress, airway epithelial injury, release of pro-inflammatory cytokines promoting type-2 inflammatory responses, and disruption of the Th17/regulatory T cell balance (21). Exposure occurs both outdoors and indoors, with some evidence that indoor concentrations of certain pollutants, particularly in poorly ventilated or high-traffic urban settings may exceed outdoor levels. Indoor PM_2.5_ and NO_2_ have been associated with asthma symptoms, particularly in children (67, 89, 90). Evidence from systematic reviews and meta-analyses supports an association between long-term PM_2.5_ and traffic-related NO_2_ exposure and incident asthma, though results for ozone and sulfur dioxide remain heterogeneous across studies (20). Among studies included in this review, Holst et al. (22) reported a significant association between air pollution exposure and asthma-related outcomes in children, further supporting the role of environmental pollutants in asthma etiology.

## 3 Genetic predisposition to asthma

Genetic predisposition is a significant and well-established risk factor for asthma. Family and twin studies demonstrate moderate-to-high disease heritability, and parental asthma increases offspring risk (91). Evidence suggests that genetic factors influence not only susceptibility but also disease severity: polymorphisms in genes such as *GSDMB* (rs7216389) and *IL18R1* have been associated with more severe clinical presentations, including greater lung function decline and higher exacerbation frequency.

Large-scale genomic studies have identified numerous susceptibility loci. Zhong et al. (92), using integrated functional genomics and fine-mapping, identified 76 genes associated with adult-onset asthma and 203 genes associated with childhood-onset asthma, including putative causal genes and candidate regulatory elements distinguishing the two phenotypes. Saarentaus et al. (93) reported 87 significantly associated loci, including newly identified loci linked to comorbid conditions such as chronic rhinosinusitis with nasal polyposis; pathway analysis implicated immune regulation and type-2 inflammatory signaling, particularly Jak-STAT pathways. Akhmerova et al. (94) analyzed 167 candidate genes and identified 11 polymorphisms influencing asthma risk, with sex-specific distributions: rs869106717 (*FOXP1*), rs1461555098 (*TACR3*), rs189649077 (*EGFR*), and rs1199362453 (*ZNF257*) were more prevalent in males; rs1923038536 (*CYSLTR1*), rs181066119 (*IL5RA*), rs143247175 (*NRG1*), rs140597386 (*HDC*), and rs762042586 (*DPP10*) were more common in females; while rs1219244986 (*MUC1*) and rs2291651 (*MUC4*) appeared to be protective in women. Variants in genes encoding adenosine receptors including *ADORA1* and *ADORA2A* have additionally been linked to aspirin-induced asthma (95).

Ferreira et al. (96) demonstrated that childhood-onset and adult-onset asthma share several risk loci but also exhibit distinct genetic architectures, pointing to both common and phenotype-specific mechanisms. Han et al. (28) identified 212 asthma-associated loci, many in immune system pathways. Earlier GWAS by Moffatt et al. (97, 98) established variants regulating *ORMDL3* expression as important contributors to childhood asthma risk, while a subsequent consortium study confirmed additional susceptibility loci including rs3771166, consistent with the complex polygenic structure of the disease. According to the Ma’ayan Lab database, more than 1, 200 genes have been reported to show some association with asthma susceptibility, among them key immune-regulatory genes such as *IL33, IL1RL1, HLA-DQA1, HLA-DQB1, ORMDL3*, and *GSDMB* (99).

### 3.1 Functional pathway overview of asthma-related genes

**Figure 2:**
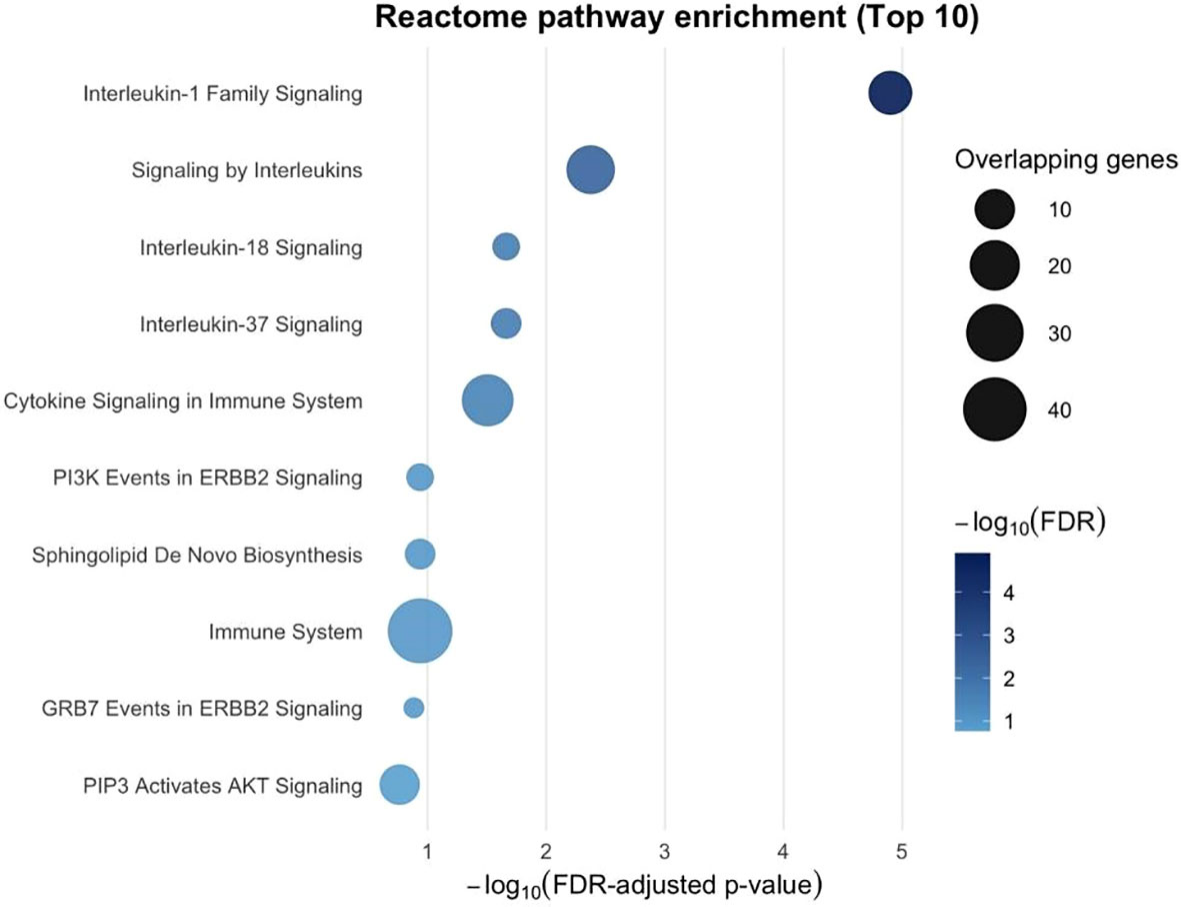
Reactome pathway enrichment analysis of the top 10 pathways. Caption: Pathway enrichment analysis using the Reactome database. The x-axis shows pathway significance (-log10 FDR-adjusted p-value), with higher values indicating stronger significance. Dot size represents the number of overlapping genes and color reflects significance level. The results show that interleukin and cytokine signaling are the most enriched, outlining the key role of immune and inflammatory processes in asthma.

To provide functional context for the genetic component of asthma, 241 asthma-associated genes were retrieved from the Ma’ayan Lab database and mapped to Reactome Pathway database entries (version 2024). This complementary mapping was used to summarize the major biological processes represented among asthma-related genes. The 10 most significantly enriched pathways, comprising 50 genes, are presented in **Figure 2**; the complete pathway overview is provided in **Supplementary S1**.

Analysis identified a cluster of genes centrally involved in regulating immune and inflammatory cascades critical to asthma pathogenesis. Key genes include *IL13, IL18R1, IL1RL1, SMAD3, STAT3, IL33, IL1RL2, NOD2, AGER, ERBB2, IL23R, IL6R, RORA, TNFSF15* and *ORMDL3*, participating in pathways including Interleukin-1 family signaling, signaling by interleukins, cytokine signaling in the immune system, IL-18/IL-37 signaling and PI3K-AKT signaling.

These gene functions are well-characterized in the asthma literature. Epithelial *IL33* activates a type-2 immune response via the ST2 receptor (*IL1RL1*), driving Th2 cytokine production including *IL13* responsible for mucus hypersecretion and bronchial hyperreactivity (100, 101). *SMAD3* links IL-1β and TGF-β signaling, mediating the transition from acute inflammation to chronic fibrosis (102). Epithelial *STAT3* regulates the intensity of allergic inflammation and Th2 cell accumulation in the lung (103). *NOD2*, as an innate sensor, participates in *IL-1β* maturation and amplification of the inflammatory response (104), while *AGER* promotes asthmatic inflammation by stimulating *IL33*-dependent activation of type-2 innate lymphoid cells (105). Additional *IL-1* family signaling axes including *IL1RL1, IL1RL2, IL23R*, and *IL18R1* are associated with neutrophilic and severe airway inflammation, helping to define disease endotypes (106–109) *RORA* is necessary for development of the Th2-mediated allergic response (110), *IL-6R* signaling correlates with disease severity and exacerbation frequency (111), the *TNFSF15* axis links inflammation to airway remodeling and tissue damage (112), and *ORMDL3* influences cytokine responses, cellular stress, and epithelial metabolic processes, contributing to a persistent inflammatory asthma phenotype (113).

A second group of genes in cluster *PI3K-AKT*, sphingolipid biosynthesis, immune system with more limited experimental links to asthma identified primarily through single GWAS includes *USP13, MECOM, GATAD2A, CERS5, SPTLC2, PRKD1, CRP* and others distributed across *PI3K-AKT*, sphingolipid biosynthesis and immune system clusters. While their roles in asthma pathophysiology have not been directly studied, they represent candidates of interest for future investigation into metabolic and signaling dysregulation in the disease.

Overall, pathway analysis confirms that genetic susceptibility to asthma is predominantly expressed through disruption of interleukin and cytokine signaling coordination, confirming the central role of immune inflammation in asthma pathogenesis and offering a functional framework for interpreting gene-environment interaction findings.

### 3.2 Genetic susceptibility to PM_2.5_ related asthma

Asthma development results from a complex interaction of environmental exposures and genetic predisposition and studying their combined effects offers the deepest insight into individual-level variation in disease risk. In that context, fine particulate matter occupies a particularly important position: PM_2.5_ is found across all world regions, carries well-documented biological effects and as the following evidence indicates, its effect on asthma risk is substantially modified by genetic background.

A literature search identified only 9 published studies examining PM_2.5_-genetic variant interactions in relation to asthma, predominantly from Europe and East Asia, with limited representation from other regions. The limited number of studies reflects how recently polygenic risk score methods have emerged, as well as the practical difficulty of collecting both genetic data and accurate PM_2.5_ exposure measurements in the same cohort. The findings discussed here therefore require validation in larger, more diverse samples before definitive conclusions can be drawn. In most studies reviewed, PM_2.5_ rarely acts as an independent risk factor for asthma *de novo*; rather, its influence on risk or disease progression is most clearly expressed in individuals carrying specific predisposing genetic variants (114–122).

The clearest and most reproducible gene-PM_2.5_ interactions identified to date involve genes in the detoxification and oxidative stress pathway, particularly *GSTP1*. A large national cross-sectional study of Taiwanese children found that the same increment of PM_2.5_ exposure produced opposite effects depending on *GSTP1* genotype: children with the Ile/Ile genotype showed a lower risk of asthma and wheezing (OR = 0.60, 95% CI 0.45-0.82), while carriers of at least one Val allele showed a higher risk (OR = 1.52, 95% CI 1.01-2.27), with a statistically significant interaction (p=0.001) (114). Because exposure levels were comparable between genotype groups, these data indicate that genetic differences change the biological response to PM_2.5_ rather than reflecting differential exposure. Other findings emerged from the multicenter Traffic, Asthma and Genetics study, in which children carrying sensitive *GSTP1* variants had more than twice the asthma odds per 4 μg/m³ rise in PM_2.5_ (OR = 2.08, 95% CI 1.35-3.19), showing that the body’s ability to detoxify pollutants shapes individual responses to PM_2.5_ (115).

Broader genetic studies, including GWAS and polygenic risk score analyses, have identified additional loci involved in PM_2.5-_asthma interactions. The rs9836522 variant was associated with a reduced risk of asthma exacerbations during higher PM_2.5_ periods (OR = 0.75, 95% CI 0.62-0.91) in cohorts of children of African descent, while children with high polygenic risk of PM_2.5_ sensitivity experienced higher exacerbation frequency during periods of poor air quality (116). Importantly, were observed mainly as exacerbations rather than incident asthma diagnosis, showing that PM_2.5_ may act to amplify disease activity in genetically predisposed individuals rather than to initiate asthma *de novo*.

The 17q12–21 locus is one of the most replicated susceptibility regions in asthma genetics, also participates in PM_2.5_ interactions. Population studies have shown that high PM_2.5_ exposure increases asthma risk both in individuals with the CC genotype and in risk-allele carriers, but the strong association was observed when genetic predisposition and high exposure co-occurred (OR = 2.20, 95% CI 1.70-2.83) (117), with a multiplicative or synergistic interaction model in which the combination of genetic predisposition and high pollution exposure carries more risk than the sum of each factor separately.

In adult populations, a large prospective cohort study found that individuals combining high PM_2.5_ levels with a high polygenic index associated with gut microbiota composition had greater asthma risk (HR = 1.13, 95% CI 1.05-1.22) (118), pointing to the fact that microbiome-mediated immune modulation is an additional axis through which genetic factors shape PM_2.5_ susceptibility. Among preterm infants, PM_2.5_ effects on exacerbations were larger: extremely and very preterm infants showed a 43.3% increase per unit PM_2.5_ rise (p=0.001), and late preterm infants with high polygenic risk experienced an additional 24% increase (p=0.04) (119), suggesting that genetic PM_2.5_ effects are strongest in the most vulnerable children.

Studies examining respiratory function and immune response as intermediate outcomes, rather than asthma diagnosis directly, help explain the biological pathways behind these associations. Panel studies have shown that short-term PM_2.5_ elevations are associated with increased *IL-6* and *TNF-α* levels, with more pronounced inflammatory responses in GSTM1-positive individuals, whereas lung function changes (FVC, PEF, MVV) were more marked in GSTM1-null individuals (120). Short-term PM_2.5_ exposure has also been associated with decreased FEV1 z-scores without concurrent DNA methylation changes (121). Gene-PM_2.5_ interactions for lung function outcomes have generally not reached statistical significance for clinical asthma endpoints (122), but this may reflect inadequate statistical power rather than a true absence of effect.

In summary, the available evidence states a model in which PM_2.5_ exposure is not a universal asthma risk factor but rather a conditionally harmful exposure whose effects are increased by specific genetic backgrounds, mostly involving detoxification capacity (*GSTP1, GSTM1*) and the 17q12–21 susceptibility locus. Effects limited to inflammatory markers or lung function measures, without translation to clinical asthma outcomes, remain difficult to interpret given the heterogeneity of study designs and outcomes. This may explain why PM_2.5-_asthma associations have varied so widely across studies and suggests that genetic background should be considered when evaluating individual environmental risk.

## 4 Conclusion

According to the studies utilized in this review, asthma risk results from the combined effects of genetic susceptibility and environmental exposures. The interaction between PM_2.5_ and genetic variants, particularly those involved in oxidative stress detoxification and the 17q12–21 locus produces risk greater than either factor alone, pointing to the need for targeted public health action. While GWAS have advanced our understanding of asthma genetics, identified variants still explain only part of disease heritability, and pathway analysis points to cytokine and interleukin signaling disruption as the central biological mechanism. Non-genetic determinants including infections, allergens, smoking, obesity, diet, stress and occupational exposures further contribute through overlapping pathways and develop cumulative effect. Overall, the evidence suggests that a healthy lifestyle, combined with reduced exposure to environmental pollutants, may play an important role in decreasing both the risk and severity of asthma.

However, the current evidence base leaves several important questions unanswered. The number of studies examining PM_2.5-_genetic interaction are geographically limited to draw reliable conclusions. Larger cohort studies combining individual exposure data with genetic profiling, with longitudinal epigenetic designs, are needed to better understand gene-environment interactions and improve asthma prevention strategies.

## Data Availability

The original contributions presented in the study are included in the article/**Supplementary Material**. Further inquiries can be directed to the corresponding authors.
