# Supplementary figures and images for: Genetic and environmental risk factors of asthma: a narrative review
Source: Front Immunol. 2026 May 28;17:1815798. doi: 10.3389/fimmu.2026.1815798 (PMC13253968; doi:10.3389/fimmu.2026.1815798)

Reactome pathway enrichment (Enrichr)

Pathways

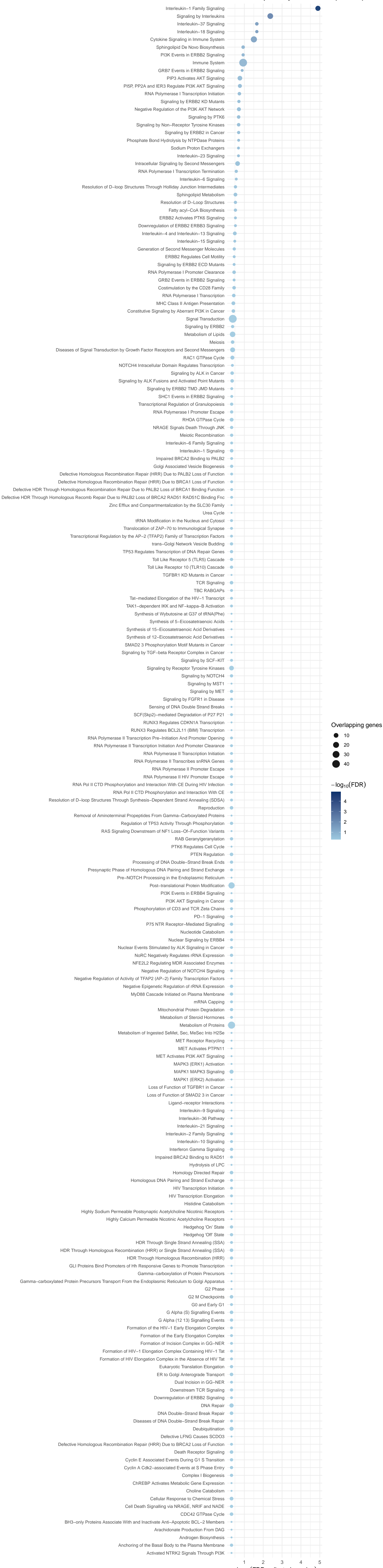

Supplement: Supplementary file 1 [file DataSheet1.pdf]
